# 7-{4-[(1,3-Benzodioxol-5-yl)meth­yl]piperazin-1-yl}-1-cyclo­propyl-6-fluoro-4-oxo-1,4-dihydro­quinoline-3-carb­oxy­lic acid

**DOI:** 10.1107/S1600536812028711

**Published:** 2012-06-30

**Authors:** Shuo Wang, Guangzhi Shan, Huiyuan Guo, Mingliang Liu

**Affiliations:** a Institute of Medicinal Biotechnology, Chinese Academy of Medical Sciences and Peking Union Medical College, Tiantan Xili 1#, Beijing, People’s Republic of China

## Abstract

In the title structure, C_25_H_24_FN_3_O_5_, a strong intra­molecular O—H⋯O hydrogen bond is present between the carb­oxy group at the 3-position and the carbonyl group at the 4-position. In the crystal, mol­ecules are held together by weak C—H⋯O, C—H⋯F and π–π [centroid–centroid distance 3.6080 (8) Å] inter­actions. The 1,4-dihydro­quinoline ring and cyclo­propyl group are not in the same plane, making an inter­planar angle of 57.52 (8)°.

## Related literature

For the synthesis and properties of quinolone derivatives, see Basuri *et al.* (2011 ▶); Feng *et al.* (2011 ▶); Guo *et al.* (2011 ▶); Liu *et al.* (2010 ▶); Sharma *et al.* (2010 ▶); Xu *et al.* (2007 ▶). For the cryogenic cooler used in the data collection, see Cosier & Glazer (1986 ▶). For hydrogen bonding, see Desiraju & Steiner (1999 ▶).
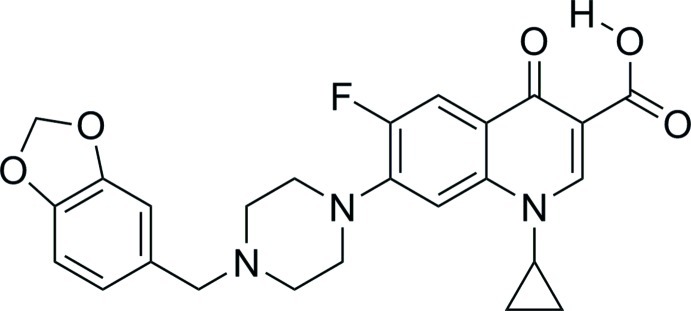


## Experimental

### 

#### Crystal data

C_25_H_24_FN_3_O_5_
*M* *_r_* = 465.47
Triclinic, 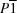

*a* = 8.6200 (5) Å
*b* = 9.7068 (9) Å
*c* = 13.7680 (13) Å
α = 79.089 (8)°
β = 76.000 (6)°
γ = 87.939 (6)°
*V* = 1097.52 (16) Å^3^
*Z* = 2
Cu *K*α radiation
μ = 0.88 mm^−1^
*T* = 118 K
0.55 × 0.35 × 0.15 mm

#### Data collection

Oxford Gemini S Ultra Sapphire CCD diffractometer
Absorption correction: multi-scan (*CrysAlis PRO*; Agilent, 2011 ▶) *T*_min_ = 0.645, *T*_max_ = 0.880
10407 measured reflections
3876 independent reflections
3518 reflections with *I* > 2σ(*I*)
*R*_int_ = 0.024

#### Refinement

*R*[*F* ^2^ > 2σ(*F* ^2^)] = 0.036
*wR*(*F* ^2^) = 0.099
*S* = 1.04
3876 reflections
311 parameters
H atoms treated by a mixture of independent and constrained refinement
Δρ_max_ = 0.25 e Å^−3^
Δρ_min_ = −0.20 e Å^−3^

### 

Data collection: *CrysAlis PRO* (Agilent, 2011 ▶); cell refinement: *CrysAlis PRO*; data reduction: *CrysAlis PRO*; program(s) used to solve structure: *SHELXS97* (Sheldrick, 2008 ▶); program(s) used to refine structure: *SHELXL97* (Sheldrick, 2008 ▶); molecular graphics: *SHELXTL* (Sheldrick, 2008 ▶); software used to prepare material for publication: *SHELXTL*, *PLATON* (Spek, 2009 ▶) and *publCIF* (Westrip, 2010 ▶).

## Supplementary Material

Crystal structure: contains datablock(s) global, I. DOI: 10.1107/S1600536812028711/fb2250sup1.cif

Structure factors: contains datablock(s) I. DOI: 10.1107/S1600536812028711/fb2250Isup2.hkl

Supplementary material file. DOI: 10.1107/S1600536812028711/fb2250Isup3.cml

Additional supplementary materials:  crystallographic information; 3D view; checkCIF report

## Figures and Tables

**Table 1:** Hydrogen-bond geometry (Å, °)

*D*—H⋯*A*	*D*—H	H⋯*A*	*D*⋯*A*	*D*—H⋯*A*
O3—H3⋯O2	0.992 (19)	1.563 (19)	2.5215 (13)	161.1 (17)
C20—H20⋯O13^i^	0.93	2.58	3.3206 (17)	137
C28—H28*a*⋯F1	0.97	2.18	2.8587 (15)	126
C32—H32*b*⋯O2^ii^	0.97	2.49	3.4144 (16)	158
C34—H34*b*⋯O3^iii^	0.97	2.44	3.3853 (18)	165

Symmetry codes: (i) 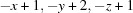; (ii) 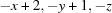; (iii) 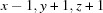.

**Fig. 1.**
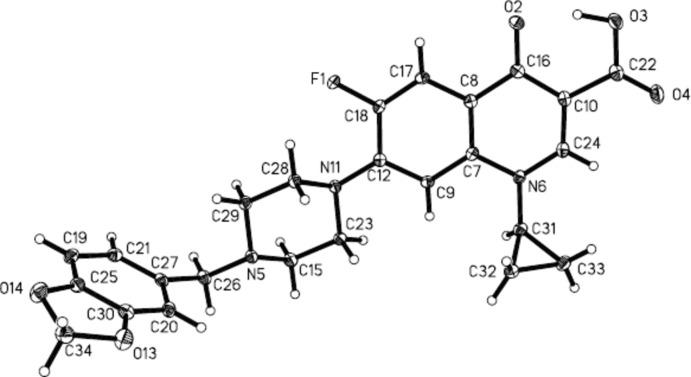
The title molecule with the atom-numbering scheme. The displacement parameters are shown at the 30% probability level.

**Fig. 2.**
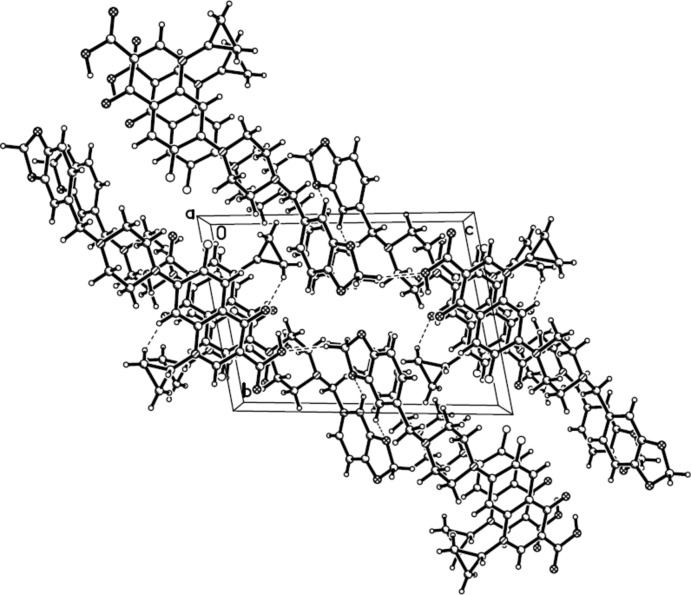
Packing of the title molecules viewed along the a direction. The dashed lines indicate the hydrogen bonds.

## supplementary crystallographic information

### Comment

Ciprofloxacin, the most applied antibacterial agent worldwide (Basuri *et al.*, 2011), is used extensively for the treatment of various bacterial infections including tuberculosis (Liu *et al.*, 2010). However, extensive use and even abuse have brought increasing ciprofloxacin resistance to many Gram-positive and Gram-negative pathogens, as well as to Mycobacterium tuberculosis (Xu *et al.*, 2007; Liu *et al.*, 2010). Recently, as a part of our program which is aimed to increase potency and to overcome resistance of existing quinolones by their structural modifications, we have focused our attention on introducing various lipophilic groups, such as an isatin or a coumarin moieties, to ciprofloxacin (Feng *et al.*, 2011; Guo *et al.*, 2011). Some of the ciprofloxacin derivatives were found to have improved activity against drug-resistant Mycobacterium tuberculosis. Our results have suggested that the activity of fluoroquinolones against drug-resistant Mycobacterium tuberculosis is proportional to increment of lipophilicity in ciprofloxacin derivatives (Sharma *et al.*, 2010).

The crystal structure of the title compound is reported here. The title compound shows remarkable improvement in lipophilicity by introduction of a lipophilic 3,4-methylenedioxyl benzyl group to the N atom which is situated on the C-7 piperazine ring of ciprofloxacin.

The title molecule is shown in Fig. 1. The 1,4-dihydroquinoline ring and cyclopropyl group (C31\C32\C33) are not in the same plane and the interplanar angle between them is 57.52 (8)°. The six-membered piperazine ring adopts a chair conformation. In the title structure, there is a strong intramolecular hydrogen bond O—H···O and a weak C-H···F interaction (Table 1; Fig. 2) (Desiraju & Steiner, 1999). The intermolecular interactions that are present in the structure are weak ones exclusively: a) C—H···O hydrogen bonds (Table 1) and b) π-electron ring — π-electron ring interactions in the structure as it is indicated by the distance 3.6080 (8) Å between the respective centroids of the benzene rings C7\C8\C17\C18\C12\C9 (symmetry codes *x*, *y*, *z* and 1*-x*, 1*-y*, *-z*).

### Experimental

To a stirred solution of piperonyl alcohol (0.61 g, 4 mmol) in anhydrous methylene chloride (50 ml) at 0–5°C was added phosphorus tribromide (0.5 ml, 5 mmol) dropwise over a period of 15 min. The reaction mixture was stirred for additional 30 min at the 0–5°C, washed with saturated brine, dried over anhydrous sodium sulfate and concentrated under reduced pressure. The obtained residue was dissolved in *N*,*N*-dimethyl formamide (20 ml) and anhydrous potassium carbonate (0.83 g, 6 mmol) with ciprofloxacin hydrochloride (0.51 g, 1.4 mmol) were added to this solution. The reaction mixture was heated to 40°C and stirred at this temperature for 14 h and then diluted with methylene chloride (50 ml), washed with distilled water (50 ml), dried over anhydrous sodium sulphate and concentrated under reduced pressure. The residue was purified by column chromatography (silica gel), eluted by methylene chloride and methanol in proportion 10:1 (*v*/*v*) to yield the title compound (0.32 g, 17.2 wt. %) as transparent block-like light yellow crystals, the longest distance of which was about 4 mm. The measured sample was cut from a larger crystal. Melting point: 238–239°C. ^1^H-NMR(DMSO, δ): 1.16–1.18 (2*H*, m, CH_2_ cyclopropyl), 1.28–1.31(2*H*, m, CH_2_cyclopropyl), 2.57–2.58 (4*H*, m, 2CH_2_ piperazinyl), 3.30–3.33 (4*H*, m, 2CH_2_ piperazinyl), 3.47 (2*H*, s, CH_2_ Benzyl), 3.78–3.82 (1*H*, m, CH cyclopropyl), 5.99(2*H*, s, OCH_2_O), 6.78–6.90(3*H*, m, CH benzyl), 7.55–7.57(1*H*, d, C8, *J*=8 Hz), 7.88–7.91(1*H*, d, C5, *J*=12 Hz),8.649(1*H*, s, C2), 15.21(1*H*, s, COOH). MS (ESI, m/*z*): 466 (*M*+H^+^). HRMS(ESI,m/*z*): C_25_H_24_FN_3_O_5_ Calculated:466.1769; Found:466.1772.

### Refinement

All the H atoms were discernible in the difference electron density map. The positional parameters of the hydrogen H3 involved in the strong hydrogen bond O—H···O (Table 1) were refined freely while its displacement parameter was constrained: *U*_iso_(H3)=1.5*U*_eq_(O3). The aryl, methine and methylene hydrogens were constrained in the riding atom approximation: C—H = 0.95, 1.0, 0.99 Å for aryl, methine and methylene H atoms, respectively, while *U*_iso_(H) = 1.2*U*_eq_(C) for aryl, methine and methylene.

### Crystal data

**Table:**

C_25_H_24_FN_3_O_5_	*Z* = 2
*M**_r_* = 465.47	*F*(000) = 488
Triclinic, *P*1	*D*_x_ = 1.409 Mg m^−^^3^
Hall symbol: -P 1	Melting point = 511–512 K
*a* = 8.6200 (5) Å	Cu *K*α radiation, λ = 1.54180 Å
*b* = 9.7068 (9) Å	Cell parameters from 6313 reflections
*c* = 13.7680 (13) Å	θ = 3.4–66.9°
α = 79.089 (8)°	µ = 0.88 mm^−^^1^
β = 76.000 (6)°	*T* = 118 K
γ = 87.939 (6)°	Block, colourless
*V* = 1097.52 (16) Å^3^	0.55 × 0.35 × 0.15 mm

### Data collection

**Table:**

Oxford Gemini S Ultra Sapphire CCD diffractometer	3876 independent reflections
Radiation source: Enhance Ultra (Cu) X-ray Source	3518 reflections with *I* > 2σ(*I*)
Graphite monochromator	*R*_int_ = 0.024
Detector resolution: 10.4713 pixels mm^-1^	θ_max_ = 67.0°, θ_min_ = 3.4°
ω scans	*h* = −8→10
Absorption correction: multi-scan (*CrysAlis PRO*; Agilent, 2011)	*k* = −11→11
*T*_min_ = 0.645, *T*_max_ = 0.880	*l* = −15→16
10407 measured reflections	

### Refinement

**Table:**

Refinement on *F*^2^	Secondary atom site location: difference Fourier map
Least-squares matrix: full	Hydrogen site location: difference Fourier map
*R*[*F*^2^ > 2σ(*F*^2^)] = 0.036	H atoms treated by a mixture of independent and constrained refinement
*wR*(*F*^2^) = 0.099	*w* = 1/[σ^2^(*F*_o_^2^) + (0.0558*P*)^2^ + 0.3141*P*] where *P* = (*F*_o_^2^ + 2*F*_c_^2^)/3
*S* = 1.04	(Δ/σ)_max_ < 0.001
3876 reflections	Δρ_max_ = 0.25 e Å^−^^3^
311 parameters	Δρ_min_ = −0.20 e Å^−^^3^
0 restraints	Extinction correction: *SHELXL97* (Sheldrick, 2008), Fc^*^=kFc[1+0.001xFc^2^λ^3^/sin(2θ)]^-1/4^
93 constraints	Extinction coefficient: 0.0056 (6)
Primary atom site location: structure-invariant direct methods	

### Special details

**Table:**

Experimental. The crystal was placed in the cold stream of an Oxford Cryosystems open-flow nitrogen cryostat (Cosier & Glazer, 1986) with a nominal stability of 0.1 K.
Geometry. All e.s.d.'s (except the e.s.d. in the dihedral angle between two l.s. planes) are estimated using the full covariance matrix. The cell e.s.d.'s are taken into account individually in the estimation of e.s.d.'s in distances, angles and torsion angles; correlations between e.s.d.'s in cell parameters are only used when they are defined by crystal symmetry. An approximate (isotropic) treatment of cell e.s.d.'s is used for estimating e.s.d.'s involving l.s. planes.
Refinement. Refinement of *F*^2^ against ALL reflections. The weighted *R*-factor *wR* and goodness of fit *S* are based on *F*^2^, conventional *R*-factors *R* are based on *F*, with *F* set to zero for negative *F*^2^. The threshold expression of *F*^2^ > σ(*F*^2^) is used only for calculating *R*-factors(gt) *etc*. and is not relevant to the choice of reflections for refinement. *R*-factors based on *F*^2^ are statistically about twice as large as those based on *F*, and *R*- factors based on ALL data will be even larger.

### Fractional atomic coordinates and isotropic or equivalent isotropic displacement parameters (Å^2^)

**Table:**

	*x*	*y*	*z*	*U*_iso_*/*U*_eq_	
F1	0.57863 (9)	0.83866 (7)	−0.04072 (5)	0.0264 (2)	
O2	0.99664 (10)	0.51558 (9)	−0.18920 (7)	0.0247 (2)	
O3	1.16469 (11)	0.30144 (10)	−0.21487 (7)	0.0289 (2)	
H3	1.115 (2)	0.395 (2)	−0.2171 (14)	0.043*	
O4	1.11852 (12)	0.10674 (10)	−0.09798 (8)	0.0362 (3)	
N5	0.25302 (12)	0.83641 (10)	0.31959 (8)	0.0191 (2)	
N6	0.75205 (12)	0.29596 (10)	0.08703 (8)	0.0192 (2)	
C7	0.71154 (14)	0.43605 (12)	0.05691 (9)	0.0177 (3)	
C8	0.79299 (14)	0.51058 (13)	−0.03849 (9)	0.0182 (3)	
C9	0.59360 (14)	0.50087 (13)	0.12175 (9)	0.0185 (3)	
H9	0.5426	0.4500	0.1851	0.022*	
C10	0.95480 (14)	0.30413 (13)	−0.06705 (10)	0.0206 (3)	
N11	0.43402 (12)	0.70790 (10)	0.15591 (8)	0.0188 (2)	
C12	0.55067 (14)	0.63977 (12)	0.09375 (9)	0.0178 (3)	
O13	0.41707 (12)	1.17463 (10)	0.53985 (8)	0.0342 (3)	
O14	0.31968 (13)	1.39049 (10)	0.47811 (8)	0.0345 (3)	
C15	0.20577 (14)	0.70110 (13)	0.30375 (10)	0.0219 (3)	
H15a	0.1285	0.7158	0.2620	0.026*	
H15b	0.1548	0.6445	0.3690	0.026*	
C16	0.92076 (14)	0.44720 (13)	−0.10431 (9)	0.0193 (3)	
C17	0.74649 (14)	0.64957 (13)	−0.06867 (9)	0.0197 (3)	
H17	0.7969	0.7009	−0.1320	0.024*	
C18	0.62876 (14)	0.70857 (12)	−0.00577 (9)	0.0194 (3)	
C19	0.14668 (15)	1.29718 (14)	0.38467 (10)	0.0253 (3)	
H19	0.1062	1.3835	0.3603	0.030*	
C20	0.26908 (14)	1.03395 (13)	0.45990 (10)	0.0230 (3)	
H20	0.3100	0.9483	0.4851	0.028*	
C21	0.10290 (15)	1.17255 (14)	0.36068 (10)	0.0229 (3)	
H21	0.0319	1.1768	0.3190	0.027*	
C22	1.08468 (15)	0.22684 (14)	−0.12629 (10)	0.0248 (3)	
C23	0.34835 (14)	0.62297 (13)	0.25215 (9)	0.0200 (3)	
H23a	0.4203	0.5994	0.2970	0.024*	
H23b	0.3119	0.5362	0.2393	0.024*	
C24	0.86751 (15)	0.23478 (13)	0.02508 (10)	0.0211 (3)	
H24	0.8892	0.1407	0.0458	0.025*	
C25	0.25223 (15)	1.28555 (13)	0.44597 (10)	0.0232 (3)	
C26	0.11331 (14)	0.90959 (13)	0.36969 (10)	0.0228 (3)	
H26a	0.0564	0.8482	0.4311	0.027*	
H26b	0.0415	0.9323	0.3246	0.027*	
C27	0.16168 (14)	1.04287 (13)	0.39686 (9)	0.0205 (3)	
C28	0.47789 (14)	0.84670 (12)	0.17178 (10)	0.0208 (3)	
H28a	0.5293	0.9037	0.1068	0.025*	
H28b	0.5528	0.8348	0.2151	0.025*	
C29	0.32998 (15)	0.91944 (13)	0.22088 (10)	0.0212 (3)	
H29a	0.3590	1.0111	0.2297	0.025*	
H29b	0.2559	0.9326	0.1770	0.025*	
C30	0.31084 (14)	1.15687 (14)	0.48255 (10)	0.0227 (3)	
C31	0.66294 (15)	0.21667 (13)	0.18405 (10)	0.0223 (3)	
H31	0.5509	0.1960	0.1879	0.027*	
C32	0.69772 (16)	0.24489 (14)	0.28023 (10)	0.0251 (3)	
H32a	0.6086	0.2430	0.3391	0.030*	
H32b	0.7815	0.3124	0.2739	0.030*	
C33	0.74586 (17)	0.10871 (14)	0.24508 (10)	0.0270 (3)	
H33a	0.8587	0.0942	0.2178	0.032*	
H33b	0.6859	0.0248	0.2829	0.032*	
C34	0.40629 (18)	1.31863 (15)	0.54924 (11)	0.0308 (3)	
H34a	0.5125	1.3593	0.5346	0.037*	
H34b	0.3511	1.3271	0.6181	0.037*	

### Atomic displacement parameters (Å^2^)

**Table:**

	*U*^11^	*U*^22^	*U*^33^	*U*^12^	*U*^13^	*U*^23^
F1	0.0320 (4)	0.0191 (4)	0.0236 (4)	0.0074 (3)	−0.0035 (3)	0.0020 (3)
O2	0.0194 (4)	0.0269 (5)	0.0244 (5)	−0.0011 (4)	−0.0001 (4)	−0.0028 (4)
O3	0.0208 (5)	0.0317 (5)	0.0315 (5)	0.0025 (4)	0.0002 (4)	−0.0081 (4)
O4	0.0323 (6)	0.0238 (5)	0.0467 (6)	0.0058 (4)	0.0024 (5)	−0.0085 (4)
N5	0.0166 (5)	0.0178 (5)	0.0217 (5)	−0.0018 (4)	−0.0008 (4)	−0.0051 (4)
N6	0.0191 (5)	0.0158 (5)	0.0227 (5)	−0.0007 (4)	−0.0058 (4)	−0.0029 (4)
C7	0.0170 (6)	0.0162 (6)	0.0221 (6)	−0.0017 (4)	−0.0083 (5)	−0.0037 (5)
C8	0.0157 (6)	0.0202 (6)	0.0208 (6)	−0.0017 (5)	−0.0069 (5)	−0.0051 (5)
C9	0.0180 (6)	0.0188 (6)	0.0183 (6)	−0.0027 (5)	−0.0042 (5)	−0.0020 (5)
C10	0.0170 (6)	0.0214 (6)	0.0258 (7)	−0.0001 (5)	−0.0069 (5)	−0.0081 (5)
N11	0.0175 (5)	0.0169 (5)	0.0210 (5)	−0.0011 (4)	−0.0021 (4)	−0.0039 (4)
C12	0.0148 (6)	0.0186 (6)	0.0214 (6)	−0.0012 (4)	−0.0062 (5)	−0.0050 (5)
O13	0.0371 (6)	0.0307 (5)	0.0432 (6)	0.0005 (4)	−0.0224 (5)	−0.0109 (4)
O14	0.0457 (6)	0.0225 (5)	0.0402 (6)	−0.0072 (4)	−0.0188 (5)	−0.0052 (4)
C15	0.0188 (6)	0.0205 (6)	0.0249 (6)	−0.0052 (5)	−0.0009 (5)	−0.0047 (5)
C16	0.0152 (6)	0.0225 (6)	0.0221 (6)	−0.0034 (5)	−0.0066 (5)	−0.0059 (5)
C17	0.0194 (6)	0.0199 (6)	0.0189 (6)	−0.0034 (5)	−0.0043 (5)	−0.0013 (5)
C18	0.0201 (6)	0.0154 (6)	0.0230 (6)	0.0007 (5)	−0.0072 (5)	−0.0016 (5)
C19	0.0260 (7)	0.0204 (6)	0.0267 (7)	−0.0003 (5)	−0.0045 (5)	0.0007 (5)
C20	0.0195 (6)	0.0216 (6)	0.0267 (7)	0.0032 (5)	−0.0029 (5)	−0.0054 (5)
C21	0.0192 (6)	0.0266 (7)	0.0216 (6)	−0.0009 (5)	−0.0036 (5)	−0.0030 (5)
C22	0.0190 (6)	0.0257 (7)	0.0312 (7)	−0.0011 (5)	−0.0054 (5)	−0.0097 (6)
C23	0.0208 (6)	0.0174 (6)	0.0208 (6)	−0.0032 (5)	−0.0027 (5)	−0.0032 (5)
C24	0.0194 (6)	0.0183 (6)	0.0280 (7)	0.0006 (5)	−0.0081 (5)	−0.0071 (5)
C25	0.0239 (6)	0.0195 (6)	0.0237 (6)	−0.0047 (5)	−0.0008 (5)	−0.0034 (5)
C26	0.0165 (6)	0.0250 (7)	0.0257 (7)	−0.0014 (5)	−0.0005 (5)	−0.0076 (5)
C27	0.0149 (6)	0.0236 (6)	0.0205 (6)	−0.0012 (5)	0.0026 (5)	−0.0062 (5)
C28	0.0188 (6)	0.0165 (6)	0.0250 (6)	−0.0028 (5)	−0.0003 (5)	−0.0045 (5)
C29	0.0203 (6)	0.0178 (6)	0.0235 (6)	−0.0006 (5)	−0.0019 (5)	−0.0034 (5)
C30	0.0176 (6)	0.0273 (7)	0.0232 (6)	−0.0015 (5)	−0.0038 (5)	−0.0058 (5)
C31	0.0216 (6)	0.0186 (6)	0.0252 (7)	−0.0034 (5)	−0.0048 (5)	−0.0003 (5)
C32	0.0270 (7)	0.0228 (6)	0.0235 (7)	−0.0004 (5)	−0.0038 (5)	−0.0020 (5)
C33	0.0339 (7)	0.0198 (6)	0.0271 (7)	0.0010 (5)	−0.0093 (6)	−0.0011 (5)
C34	0.0354 (8)	0.0301 (7)	0.0292 (7)	−0.0067 (6)	−0.0087 (6)	−0.0083 (6)

### Geometric parameters (Å, º)

**Table:**

F1—C18	1.3580 (14)	C17—C18	1.3522 (17)
O2—C16	1.2657 (15)	C19—H19	0.9300
O3—H3	0.992 (19)	C19—C21	1.3998 (19)
O3—C22	1.3349 (17)	C19—C25	1.3714 (19)
O4—C22	1.2061 (17)	C20—H20	0.9300
N5—C15	1.4590 (15)	C20—C27	1.4044 (18)
N5—C26	1.4673 (15)	C20—C30	1.3700 (18)
N5—C29	1.4575 (16)	C21—H21	0.9300
N6—C7	1.4020 (16)	C21—C27	1.3886 (18)
N6—C24	1.3451 (16)	C23—H23a	0.9700
N6—C31	1.4573 (16)	C23—H23b	0.9700
C7—C8	1.4043 (17)	C24—H24	0.9300
C7—C9	1.3978 (18)	C25—C30	1.3816 (18)
C8—C16	1.4464 (17)	C26—H26a	0.9700
C8—C17	1.4082 (17)	C26—H26b	0.9700
C9—H9	0.9300	C26—C27	1.5116 (17)
C9—C12	1.3927 (17)	C28—H28a	0.9700
C10—C16	1.4324 (18)	C28—H28b	0.9700
C10—C22	1.4870 (18)	C28—C29	1.5100 (16)
C10—C24	1.3710 (18)	C29—H29a	0.9700
N11—C12	1.3896 (16)	C29—H29b	0.9700
N11—C23	1.4620 (15)	C31—H31	0.9800
N11—C28	1.4812 (15)	C31—C32	1.5013 (18)
C12—C18	1.4199 (17)	C31—C33	1.4895 (18)
O13—C30	1.3794 (16)	C32—H32a	0.9700
O13—C34	1.4256 (17)	C32—H32b	0.9700
O14—C25	1.3817 (16)	C32—C33	1.5031 (18)
O14—C34	1.4356 (18)	C33—H33a	0.9700
C15—H15a	0.9700	C33—H33b	0.9700
C15—H15b	0.9700	C34—H34a	0.9700
C15—C23	1.5182 (17)	C34—H34b	0.9700
C17—H17	0.9300		
			
C22—O3—H3	103.9 (10)	C15—C23—H23a	109.4
C15—N5—C26	110.56 (9)	C15—C23—H23b	109.4
C29—N5—C15	108.42 (9)	H23a—C23—H23b	108.0
C29—N5—C26	110.61 (10)	N6—C24—C10	123.29 (12)
C7—N6—C31	119.05 (10)	N6—C24—H24	118.4
C24—N6—C7	120.11 (11)	C10—C24—H24	118.4
C24—N6—C31	120.80 (10)	C19—C25—O14	128.82 (12)
N6—C7—C8	118.99 (11)	C19—C25—C30	121.38 (12)
C9—C7—N6	120.33 (11)	C30—C25—O14	109.78 (11)
C9—C7—C8	120.67 (11)	N5—C26—H26a	109.3
C7—C8—C16	121.34 (11)	N5—C26—H26b	109.3
C7—C8—C17	118.00 (11)	N5—C26—C27	111.40 (10)
C17—C8—C16	120.65 (11)	H26a—C26—H26b	108.0
C7—C9—H9	119.2	C27—C26—H26a	109.3
C12—C9—C7	121.58 (11)	C27—C26—H26b	109.3
C12—C9—H9	119.2	C20—C27—C26	118.66 (11)
C16—C10—C22	121.43 (11)	C21—C27—C20	119.83 (12)
C24—C10—C16	120.38 (11)	C21—C27—C26	121.52 (12)
C24—C10—C22	118.19 (12)	N11—C28—H28a	109.7
C12—N11—C23	116.32 (10)	N11—C28—H28b	109.7
C12—N11—C28	116.83 (9)	N11—C28—C29	109.89 (9)
C23—N11—C28	110.59 (9)	H28a—C28—H28b	108.2
C9—C12—C18	115.93 (11)	C29—C28—H28a	109.7
N11—C12—C9	123.34 (11)	C29—C28—H28b	109.7
N11—C12—C18	120.68 (11)	N5—C29—C28	110.38 (10)
C30—O13—C34	105.55 (10)	N5—C29—H29a	109.6
C25—O14—C34	105.13 (10)	N5—C29—H29b	109.6
N5—C15—H15a	109.3	C28—C29—H29a	109.6
N5—C15—H15b	109.3	C28—C29—H29b	109.6
N5—C15—C23	111.63 (10)	H29a—C29—H29b	108.1
H15a—C15—H15b	108.0	O13—C30—C25	109.77 (11)
C23—C15—H15a	109.3	C20—C30—O13	127.45 (12)
C23—C15—H15b	109.3	C20—C30—C25	122.75 (12)
O2—C16—C8	121.28 (11)	N6—C31—H31	115.7
O2—C16—C10	122.92 (11)	N6—C31—C32	118.35 (10)
C10—C16—C8	115.81 (11)	N6—C31—C33	119.73 (11)
C8—C17—H17	119.9	C32—C31—H31	115.7
C18—C17—C8	120.14 (11)	C33—C31—H31	115.7
C18—C17—H17	119.9	C33—C31—C32	60.34 (9)
F1—C18—C12	118.11 (10)	C31—C32—H32a	117.8
C17—C18—F1	118.36 (11)	C31—C32—H32b	117.8
C17—C18—C12	123.49 (11)	C31—C32—C33	59.44 (8)
C21—C19—H19	121.7	H32a—C32—H32b	115.0
C25—C19—H19	121.7	C33—C32—H32a	117.8
C25—C19—C21	116.60 (12)	C33—C32—H32b	117.8
C27—C20—H20	121.5	C31—C33—C32	60.22 (9)
C30—C20—H20	121.5	C31—C33—H33a	117.7
C30—C20—C27	117.07 (12)	C31—C33—H33b	117.7
C19—C21—H21	118.8	C32—C33—H33a	117.7
C27—C21—C19	122.37 (12)	C32—C33—H33b	117.7
C27—C21—H21	118.8	H33a—C33—H33b	114.9
O3—C22—C10	114.69 (11)	O13—C34—O14	108.06 (10)
O4—C22—O3	121.37 (12)	O13—C34—H34a	110.1
O4—C22—C10	123.93 (12)	O13—C34—H34b	110.1
N11—C23—C15	111.08 (10)	O14—C34—H34a	110.1
N11—C23—H23a	109.4	O14—C34—H34b	110.1
N11—C23—H23b	109.4	H34a—C34—H34b	108.4
			
N5—C15—C23—N11	−55.89 (14)	C19—C25—C30—O13	178.54 (11)
N5—C26—C27—C20	−57.52 (15)	C19—C25—C30—C20	0.5 (2)
N5—C26—C27—C21	122.24 (12)	C21—C19—C25—O14	177.31 (12)
N6—C7—C8—C16	−2.09 (17)	C21—C19—C25—C30	−0.51 (19)
N6—C7—C8—C17	177.82 (10)	C22—C10—C16—O2	0.78 (18)
N6—C7—C9—C12	−179.86 (10)	C22—C10—C16—C8	−179.11 (10)
N6—C31—C32—C33	−109.95 (13)	C22—C10—C24—N6	177.03 (11)
N6—C31—C33—C32	107.70 (13)	C23—N11—C12—C9	−3.85 (16)
C7—N6—C24—C10	2.42 (18)	C23—N11—C12—C18	173.30 (10)
C7—N6—C31—C32	−75.97 (14)	C23—N11—C28—C29	−56.22 (13)
C7—N6—C31—C33	−146.14 (11)	C24—N6—C7—C8	0.10 (17)
C7—C8—C16—O2	−178.28 (10)	C24—N6—C7—C9	−178.88 (11)
C7—C8—C16—C10	1.61 (17)	C24—N6—C31—C32	106.58 (13)
C7—C8—C17—C18	1.25 (17)	C24—N6—C31—C33	36.42 (17)
C7—C9—C12—N11	179.93 (11)	C24—C10—C16—O2	−179.31 (11)
C7—C9—C12—C18	2.66 (17)	C24—C10—C16—C8	0.80 (17)
C8—C7—C9—C12	1.18 (18)	C24—C10—C22—O3	−179.72 (11)
C8—C17—C18—F1	−174.85 (10)	C24—C10—C22—O4	−0.3 (2)
C8—C17—C18—C12	2.83 (19)	C25—O14—C34—O13	12.86 (14)
C9—C7—C8—C16	176.89 (10)	C25—C19—C21—C27	0.29 (18)
C9—C7—C8—C17	−3.21 (17)	C26—N5—C15—C23	−179.89 (10)
C9—C12—C18—F1	172.94 (10)	C26—N5—C29—C28	177.45 (10)
C9—C12—C18—C17	−4.75 (18)	C27—C20—C30—O13	−177.87 (12)
N11—C12—C18—F1	−4.41 (17)	C27—C20—C30—C25	−0.16 (18)
N11—C12—C18—C17	177.90 (11)	C28—N11—C12—C9	129.76 (12)
N11—C28—C29—N5	60.54 (13)	C28—N11—C12—C18	−53.09 (15)
C12—N11—C23—C15	−169.84 (10)	C28—N11—C23—C15	53.80 (13)
C12—N11—C28—C29	167.66 (10)	C29—N5—C15—C23	58.71 (13)
O14—C25—C30—O13	0.34 (14)	C29—N5—C26—C27	−65.90 (13)
O14—C25—C30—C20	−177.73 (11)	C30—O13—C34—O14	−12.70 (14)
C15—N5—C26—C27	174.00 (10)	C30—C20—C27—C21	−0.06 (17)
C15—N5—C29—C28	−61.18 (12)	C30—C20—C27—C26	179.70 (11)
C16—C8—C17—C18	−178.84 (11)	C31—N6—C7—C8	−177.36 (10)
C16—C10—C22—O3	0.20 (17)	C31—N6—C7—C9	3.66 (16)
C16—C10—C22—O4	179.57 (12)	C31—N6—C24—C10	179.84 (11)
C16—C10—C24—N6	−2.89 (19)	C34—O13—C30—C20	−174.34 (13)
C17—C8—C16—O2	1.81 (18)	C34—O13—C30—C25	7.71 (14)
C17—C8—C16—C10	−178.30 (11)	C34—O14—C25—C19	173.81 (13)
C19—C21—C27—C20	−0.01 (18)	C34—O14—C25—C30	−8.17 (14)
C19—C21—C27—C26	−179.77 (11)		

### Hydrogen-bond geometry (Å, º)

**Table:**

*D*—H···*A*	*D*—H	H···*A*	*D*···*A*	*D*—H···*A*
O3—H3···O2	0.992 (19)	1.563 (19)	2.5215 (13)	161.1 (17)
C20—H20···O13^i^	0.93	2.58	3.3206 (17)	137
C28—H28*a*···F1	0.97	2.18	2.8587 (15)	126
C32—H32*b*···O2^ii^	0.97	2.49	3.4144 (16)	158
C34—H34*b*···O3^iii^	0.97	2.44	3.3853 (18)	165

Symmetry codes: (i) −*x*+1, −*y*+2, −*z*+1; (ii) −*x*+2, −*y*+1, −*z*; (iii) *x*−1, *y*+1, *z*+1.
